# A comprehensive analysis of the FOX family for predicting kidney renal clear cell carcinoma prognosis and the oncogenic role of FOXG1

**DOI:** 10.18632/aging.204448

**Published:** 2022-12-29

**Authors:** Wenjie Yang, Hualin Chen, Lin Ma, Jie Dong, Mengchao Wei, Xiaoqiang Xue, Yingjie Li, Zhaoheng Jin, Weifeng Xu, Zhigang Ji

**Affiliations:** 1 Department of Urology, Peking Union Medical College Hospital, Chinese Academy of Medical Sciences and Peking Union Medical College, Dongcheng, Beijing 100000, China

**Keywords:** KIRC, prognosis prediction, FOX gene family, nomogram, immunotherapy, FOXG1

## Abstract

Previous studies have confirmed that the forkhead box (FOX) superfamily of transcription factors regulates tumor progression and metastasis in multiple cancer. The purpose of this study was to develop a model based on FOX family genes for predicting kidney renal clear cell carcinom (KIRC) prognosis. We downloaded the transcriptional profiles and clinical data of KIRC patients from the Cancer Genome Atlas (TCGA) and International Cancer Genome Consortium (ICGC) datasets. To build a new prognosis model, we screened prognosis-related FOX family genes using Lasso regression and Multivariate Cox regression analyses. Receiver operating characteristic (ROC) curves were used to evaluate model performance. Additionally, a prognostic nomogram was developed using clinical information and selected genes to improve the accuracy of prognostic prediction. We also investigated whether prognosis-related FOX family genes are related to the immune response in KIRC. Finally, we validated the oncogenic role of FOXG1 in KIRC using an *in vitro* tumor function assay. Six prognosis-related FOX family genes were screened: FOXO1, FOXM1, FOXK2, FOXG1, FOXA1, and FOXD1. The ROC curves indicated that our model was capable of making accurate predictions for 1-, 3-, and 5-year overall survival (OS). The nomogram further improved the accuracy of prognostic predictions. In addition, compared to those in patients with low-risk scores, high-risk scores predicted a decreased level of immune cell infiltration and a lower immune response rate. Moreover, the results of *in vitro* studies confirmed that FOXG1 supports the proliferation and invasion of KIRC.

## INTRODUCTION

The incidence and mortality of renal cell carcinoma (RCC) are on the rise, making it one of the most common genitourinary cancers worldwide [1]. KIRC remains the most prevalent histological subtype of renal cancer (accounting for 80–85%). Approximately 25–30% of patients have metastasis at the time of diagnosis, and over 20% of patients develop metastases after curative surgery [2]. Although great progress has been made in the diagnosis and treatment of KIRC over the past decades, the overall survival (OS) rate remains unsatisfactory, especially for metastatic KIRC, with a 5-year survival rate of less than 20% [3]. Targeted therapy has opened a new era for oncological therapies [4, 5]. However, the issue of drug resistance has also gradually come to the forefront, with most patients developing secondary drug resistance within 1 year, and no prolonged progression-free survival has been observed in patients with advanced KIRC [6, 7]. These harsh realities have pushed us to research the pathogenesis of KIRC and new therapeutic targets. Moreover, it has become increasingly important to construct a prognostic model that can accurately predict patient prognosis and allow for the formulation of effective treatment strategies.

The forkhead box (FOX) superfamily of transcription factors comprises at least 49 members [8]. These proteins are characterized by a forkhead domain that contains a ~100-amino acid monomeric domain for DNA binding [9]. As conserved transcription factors, FOX family genes play a vital role in regulating diverse biological processes, including tumor metabolism, proliferation, migration, and invasion. Different FOX transcription factors play complicated roles by activating or suppressing their target genes. For instance, FOXH and FOXK are oncogenic, while a few FOX genes function as tumor suppressors, including FOXL and FOXD3. A special focus should be placed on some FOX members, as some FOX proteins such as FOXJ, FOXO, and FOXP3 may function differently or contradictorily depends on the type of cancer [10–12]. Given that they play an important role in tumor development, we hypothesized that they could serve as potential prognostic biomarkers for KIRC.

In this study, the data from TCGA were classified into training and internal test cohorts. A comprehensive analysis of the FOX family genes in KIRC was performed using LASSO regression and multivariate Cox regression analyses. We then constructed a prognostic model using the prognosis-related FOX family genes. The accuracy and specificity of this model were validated in internal and external testing (ICGC) cohorts. In addition, we established a nomogram to improve the accuracy of the prognostic prediction for KIRC by combining clinical information and prognosis-related FOX family genes. The correlation between prognosis-related FOX family genes and immune response was also evaluated. Finally, we explored the function of a prognosis-related FOX family gene, FOXG1, in KIRC using *in vitro* experiments.

## MATERIALS AND METHODS

### Study datasets

An analysis of the transcriptome profiles and clinical information of 539 KIRC and 78 adjacent normal tissues was downloaded from TCGA database (https://portal.gdc.cancer.gov/). Using a random ratio of 7:3, all TCGA cohorts were divided into training and internal testing cohorts. In addition, information about the clinical data and gene expression of 91 KIRC patients was downloaded from the ICGC cohort and used as the external testing cohort (https://dcc.icgc.org/projects).

### Identification of differentially expressed genes (DEGs)

Differential expression analysis was performed on 533 ccRCC and 78 control samples using the limma package in R version 4.1.2. Significant DEGs were defined with a cut-off value of log2|fold change| >2 and *p*-value < 0.05.

### Construction and validation of gene risk models

In a random ratio of 7:3, patients in TCGA datasets were divided into training and internal testing cohorts. The prognostic role of these DEGs was assessed using univariate Cox regression analysis. Based on these prognosis-related DEGs, LASSO regression analysis was employed to select a panel of genes using “Glmnet” packages in R software. Subsequently, the coefficients of these candidate genes were calculated using multivariate Cox proportional hazards regression analysis, and a risk model was constructed. The risk score was calculated based on the expression and coefficient of expression of the candidate genes. According to the mean risk scores, KIRC patients were further divided into high-risk and low-risk groups. By using R version 3.4.2’s “rms” package, the nomogram model was constructed based on the final Cox proportional hazard regression model. Receiver operating characteristic (ROC) curves were used to estimate the model accuracy by calculating the area under the curve (AUC). Higher AUC values indicate better accuracy of the predictive model.

### Construction and validation of a predictive nomogram

The “survival” and “rms” packages were used to establish a nomogram that incorporates risk scores and clinical variables. ROC curves were constructed to evaluate the predictive accuracy at 1, 3, and 5 years. The model fit was evaluated using calibration plots.

### Immune cell infiltration analysis

Cell-type identification by estimating relative subsets of RNA transcripts (CIBERSORT) is a deconvolution method that quantifies 22 human hematopoietic cell phenotypes based on tissue gene expression profiles [13]. A *P*-value less than 0.05 was used to filter out 22 types of immune cells in the high- and low-risk groups.

### TIDE and MSI score calculation

Tumor immune dysfunction and exclusion (TIDE) scores are often used to predict patient response to immunotherapy. A higher TIDE score indicates that the tumor may be able to evade the immune system, while microsatellite instability (MSI) scores are positively correlated with immune response. The scores for TIDE and MSI were calculated online as previously described [14, 15].

### Clinical specimens

We collected 82 ccRCC specimens from 98 patients who underwent partial nephrectomy or radical nephrectomy at the Peking Union Medical College Hospital between 2018 and 2020. Study participants who met the following criteria were included: (1) initially diagnosis of ccRCC and no preoperative treatment history; (2) no distant metastases before surgery; and (3) Partial nephrectomy or radical nephrectomy. We collected small sections of ccRCC tissues and adjacent tissues, immediately frozen them, and embedded them in paraffin wax. The study protocol was approved by the Research Ethics Committee of the Peking Union Medical College Hospital (Beijing, China) and conducted in accordance with the ethical guidelines outlined in the Declaration of Helsinki. For the use of human tissue samples, informed consent forms were obtained from each patient or their relatives.

### Cell culture

786-O and ACHN cells were purchased from Cell Bank in Chinese Academy of Sciences (Shanghai, China). Cells were cultured in a humidified 5% CO_2_ environment at 37°C using 10% FBS DMEM (100 units/mL penicillin, 100 g/mL streptomycin).

### Quantitative real-time PCR (qRT-PCR)

In accordance with standard RNA extraction procedures, total RNA was extracted with Trizol reagent. Total RNA was converted into complementary DNA (cDNA) using the PrimeScript RT Reagent kit (Vazyme Biotech Co., Ltd., Nanjing, China). The RNA expression levels of the genes of interest were measured using a Bio-Rad CFX96 system with SYBR Green. We listed the prime sequences as follows: FOXG1 forward (5′-CTG CTT CCA GAT GAA AAC TTC AG-3′) and reverse (5′-GGC ATC GGA CTA TTT TCA CAG G-3′); GAPDH forward (5′-GGA GCG AGA TCC CTC CAA AAT-3′) and reverse (5′-GGC TGT TGT CAT ACT TCT CAT GG-3′).

### Western blot analysis

Following the previous description [16], western blot analysis was carried out using the following primary antibodies: FOXG1 (ab196868, Abcam, Cambridge, UK) and GAPDH (ab8245, Abcam, Cambridge, UK).

### FOXG1 knockdown and overexpression

We generated FOXG1 expression vectors by cloning full-length cDNA into the pCDNA3.1 (+) vector. Small interfering RNA (siRNA) specific for FOXG1 was synthesized by GenePharma Co., Ltd. (Shanghai, China), and a nonspecific duplex oligonucleotide was used as a negative control. Western blotting and qRT-PCR were used to measure FOXG1 expression.

### Cell proliferation and colony formation capability

According to previous studies, the cell-counting kit 8 (CCK-8) assay was used to evaluate cell proliferation. For the colony formation assay, we incubated 2000 cells for 14 days in a 60-mm dish. The cell colonies were fixed with paraformaldehyde for 20 min, washed twice with PBS, stained with 0.5% crystal violet (Sigma-Aldrich Corporation, St. Louis, MO, USA) for 15 min, and counted.

### Wound healing assay

Cells were seeded on six-well plates, cultured overnight, scraped with a sterile 200 μL pipette tip, and washed three times with PBS. After scratching, photographs were taken at 0 and 24 h. Three positions were measured for each well to determine the area covered by scratching.

### Transwell assay

Cell invasion assays were performed in polycarbonate membrane filters with pore sizes of 8 μm coated with Matrigel Transwell chambers (Corning, NY, USA). Ten-thousand cells were added to the upper chamber. Photographs were obtained after 24 h.

### Statistical analysis

All bioinformatics analyses and machine learning algorithms were performed using R software (version 4.1.2). The overall survival of the high-risk and low-risk groups was compared using Kaplan-Meier survival curve analysis and a log-rank test. Statistical significance was set at *P* < 0.05.

### Data availability statement

The datasets presented in this study can be found in online repositories. The names of the repository/repositories and accession number(s) can be found in the article.

## RESULTS

### Identification of prognosis-related FOX family genes in KIRC

**Figure 1:**
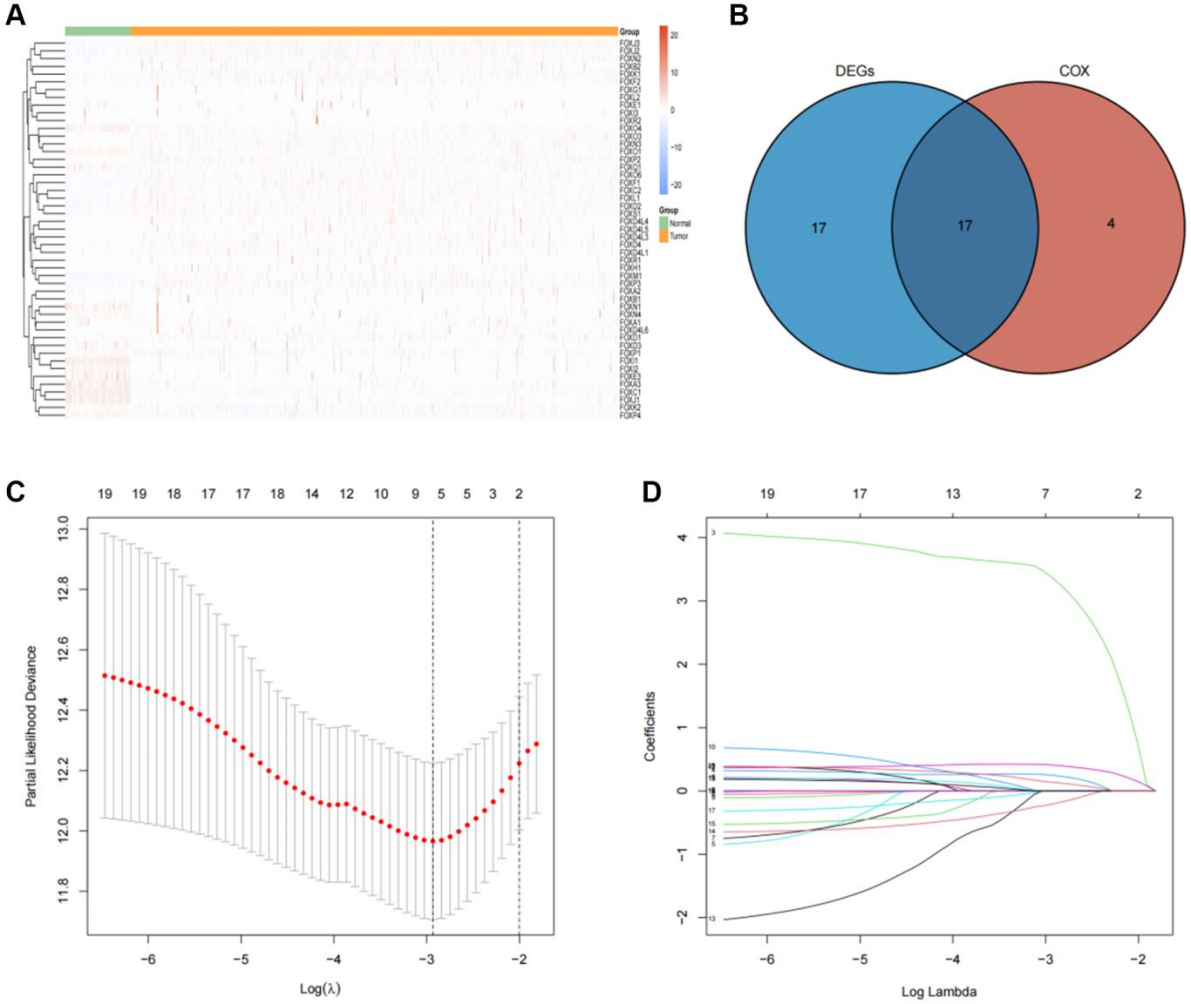
**Identification of prognosis-related FOX family genes in KIRC.** (**A**) FOX family genes expression profiles in tumors and adjacent normal tissues. (**B**) A Venn plot showing the differential expression of prognosis-related FOX family gens in KIRC tissues and adjacent non-tumor tissues. (**C**) Profiles of prognosis-related FOX family genes using Lasso coefficients. (**D**) Indicators of deviance and logarithms (lambdas).

Among the 49 FOX family genes, 34 genes demonstrated significant differential expression levels between tumors and adjacent tissues using the adjusted *p*-value < 0.05 and log FC (fold change) >2 threshold (Figure 1A, 1B). Univariate Cox regression analysis and screening of differentially expressed FOX genes revealed that 17 genes were significantly differentially expressed and strongly associated with KIRC prognosis (Figure 1B). 5 of these 17 genes were protective (HR <1), and 12 of these 17 genes were risky genes (HR >1) (Supplementary Table 1).

### Establishment of prognosis model based on six FOX family genes in the training cohort

**Figure 2:**
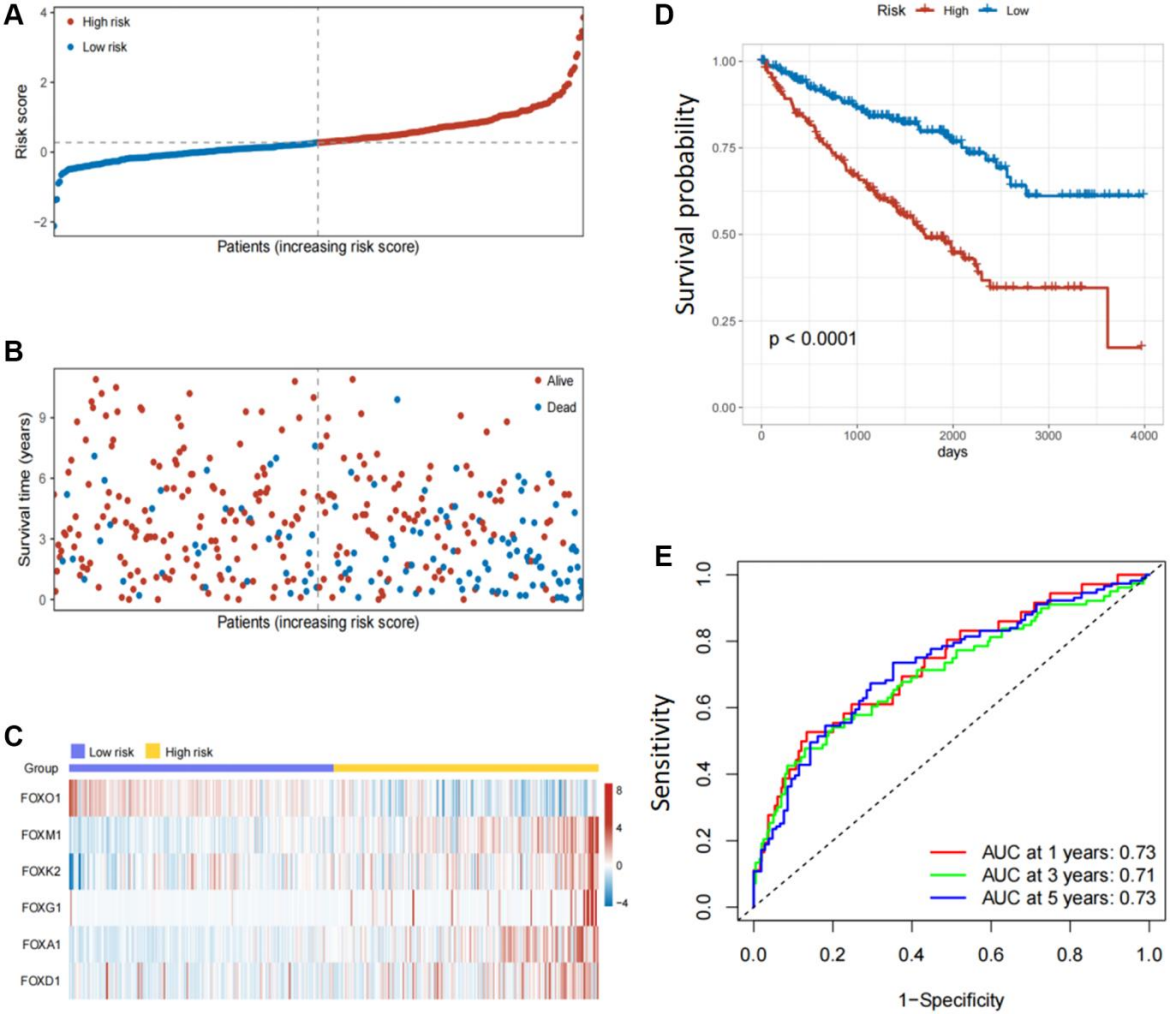
**Establishment of prognosis model based on six FOX family genes in the training cohort.** (**A**) KIRC patients’ risk scores in the training cohorts. (**B**) KIRC patients’ survival times in the training cohorts. (**C**) Correlation between the risk scores and six FOX family genes expression profile. (**D**) Survival analysis with Kaplan-Meier in the training cohorts. (**E**) AUC values of model at 1, 3, and 5 year OS in the training cohorts.

LASSO regression analysis was conducted to screen for FOX family genes that were significantly associated with KIRC patient prognosis. Minimum partial likelihood deviance was used to determine the optimal lambda value. The model was trained using 10-fold cross-validation in the training cohort (Figure 1C, 1D). As a result, we developed a prognostic model based on six FOX family genes namely, FOXO1, FOXM1, FOXK2, FOXG1, FOXA1, and FOXD1. Multivariate Cox proportional hazards regression analysis was used to calculate the Cox coefficients for the six FOX family genes. Risk scores were calculated based on the coefficients and expression levels of six FOX family genes: risk score = (−0.5478 × FOXO1) + (0.4943 × FOXM1) + (0.3012 × FOXK2) + (0.3691 × FOXG1) + (0.6456 × FOXA1) + (0.066 × FOXD1). In the training cohort, each patient's risk score was calculated. The patients were divided into high- and low-risk groups based on their median risk scores (Figure 2A). Figure 2B depicts the patients’ survival status and survival duration in the high-risk and low-risk groups. The expression profiles of the six FOX family genes in the training cohort are shown in Figure 2C. There was a lower mortality rate and better survival among patients in the low-risk group (Figure 2D). To evaluate the accuracy of this prognostic model, ROC curves for 1-, 3-, and 5-year OS were constructed. The AUC values of this model for predicting 1-, 3-, and 5-year OS were 0.73, 0.71, and 0.73, respectively (Figure 2E).

### Validation of the six FOX family genes prognosis model

**Figure 3:**
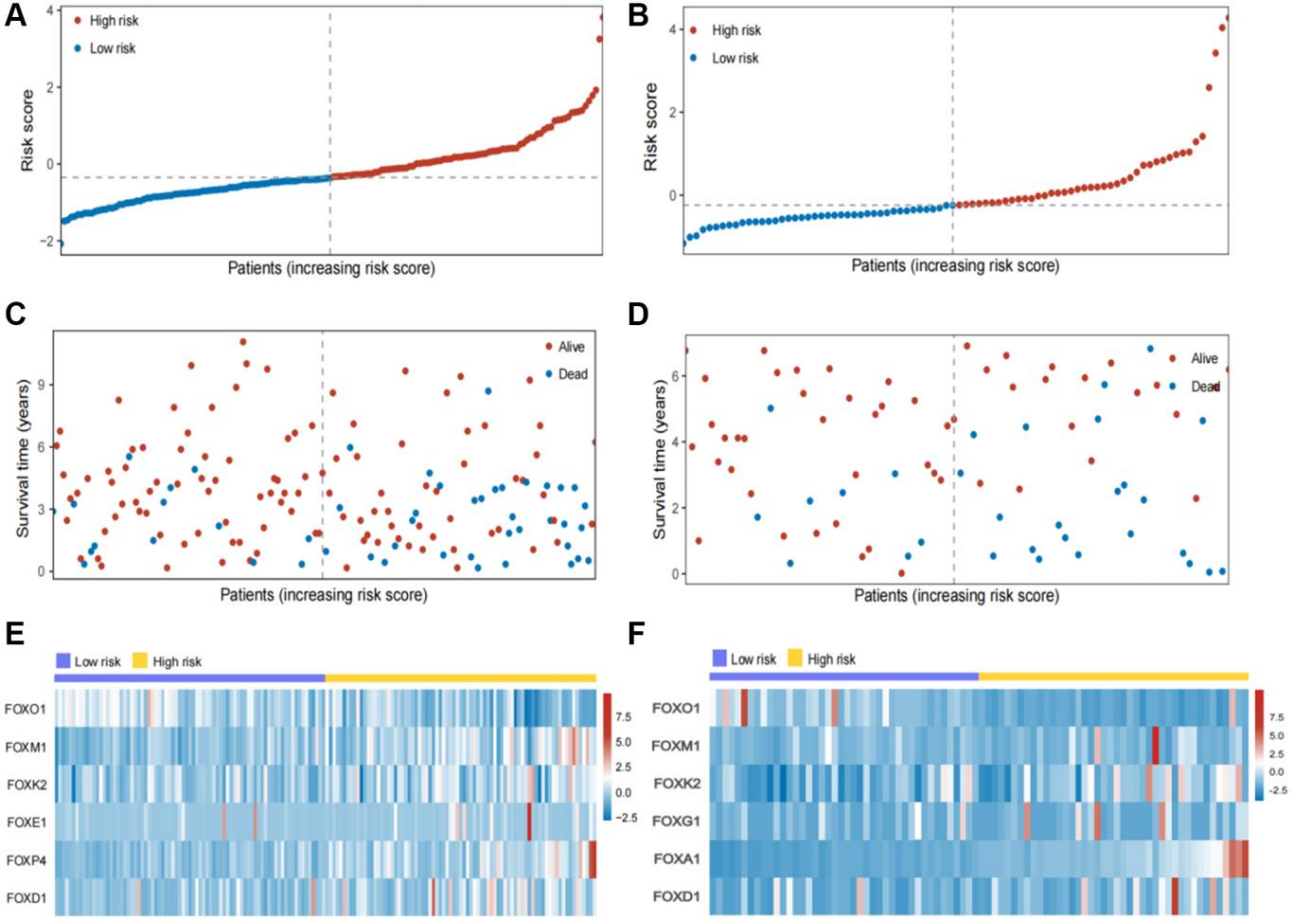
**Validation of the six FOX family genes prognosis model.** (**A**, **B**) KIRC patients’ risk scores in the internal and external testing cohorts. (**C**, **D**) KIRC patients’ risk scores in the internal and external testing cohorts. (**E**, **F**) Correlation between the risk scores and six FOX family genes expression profile.

**Figure 4:**
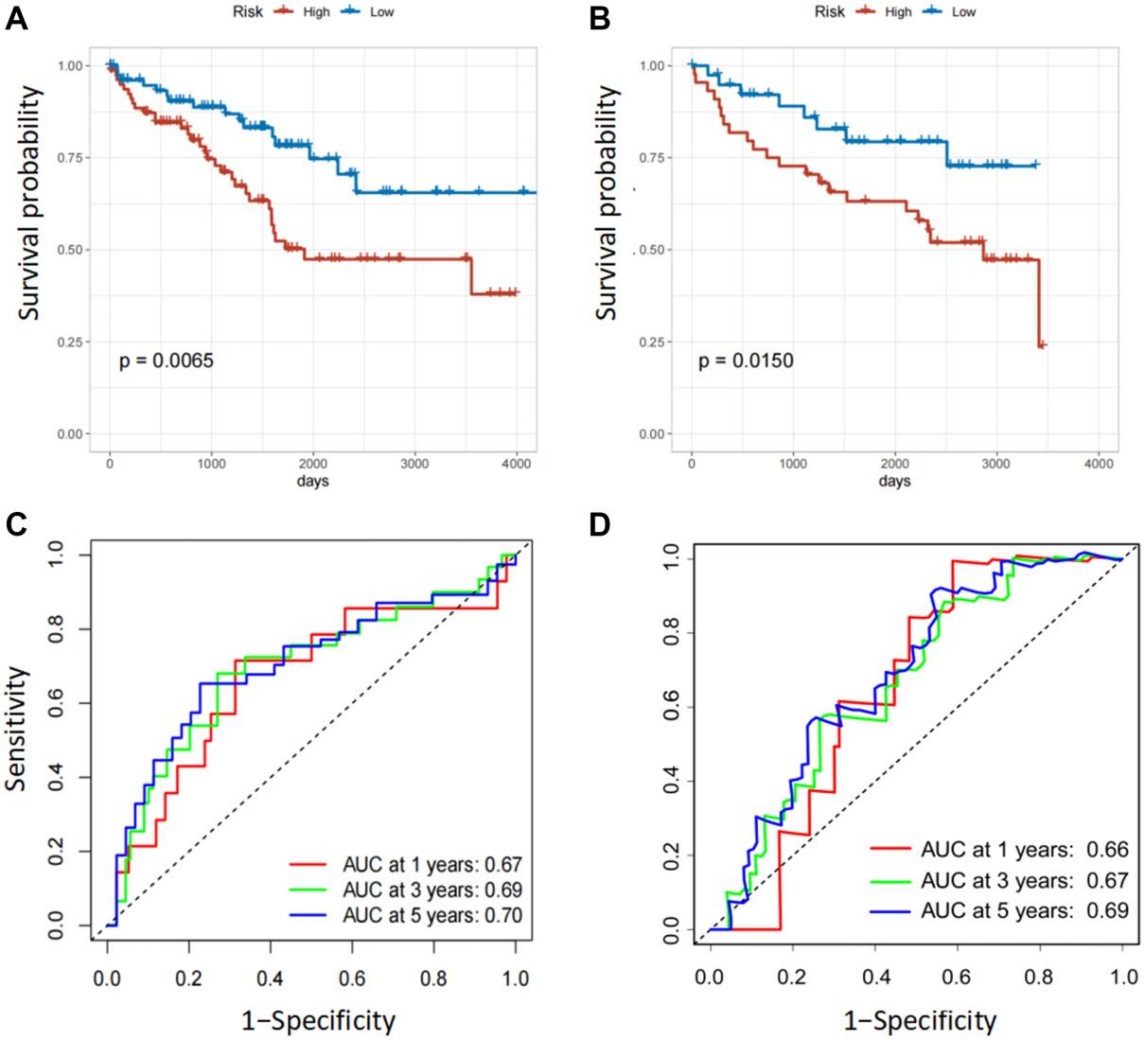
**Validation of the six FOX family genes prognosis model.** (**A**, **B**) Survival analysis with Kaplan-Meier in the internal and external testing cohorts. (**C**, **D**) AUC values of model at 1, 3, and 5 year OS in the internal and external testing cohorts.

In the internal and external testing cohorts, the same algorithm was used to calculate the patients' risk scores and divide them into high- and low-risk groups (Figure 3A, 3B). The survival rate was lower in high-risk groups (Figure 3C, 3D). The expression profiles of six FOX family genes in the internal and external testing cohorts indicated that FOXM1, FOXK2, FOXG1, FOXA1 and FOXD1exhibited higher expression in the high-risk group (Figure 3E, 3F). Patients in the low-risk group had better OS than those in the high-risk group, based on Kaplan-Meier curves (Figure 4A, 4B). The AUC values of this model for the prediction of 1-, 3-, and 5-year OS were 0.67, 0.69, and 0.70, respectively, in the internal test cohort, and 0.66, 0.67, and 0.69 in the external test cohort (Figure 4C, 4D).

### Construction of a six FOX family genes prognosis model-based nomogram

**Figure 5:**
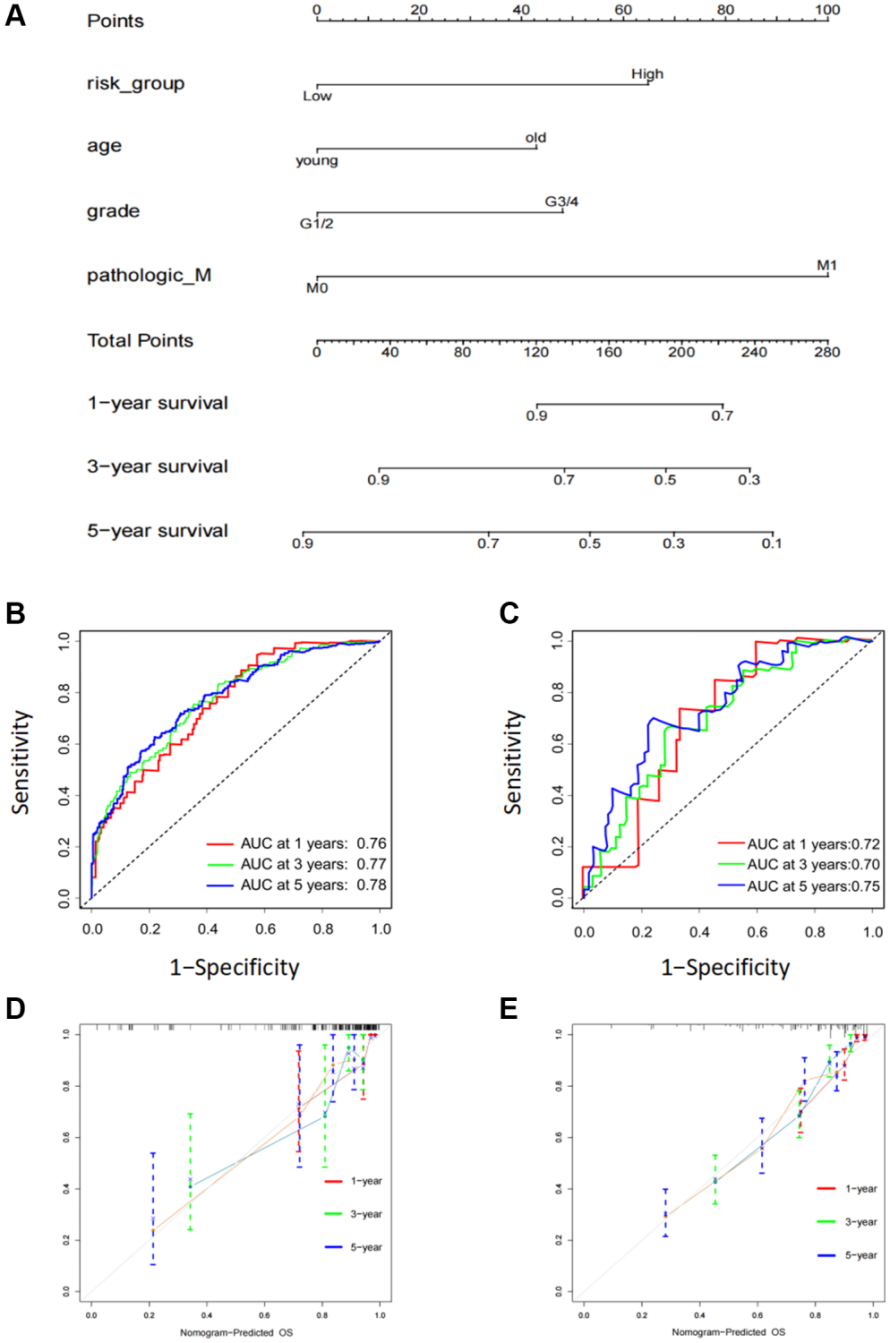
**Construction of a six FOX family genes prognosis model-based nomogram.** (**A**) A nomogram composed of clinicopathological factors including age, gender, age, M stage, as well as risk scores. (**B**) AUC values of nomogram at 1, 3, and 5 year OS in the TCGA cohorts. (**C**) AUC values of nomogram at 1, 3, and 5 year OS in the ICGC cohorts. (**D**) The calibration curves of nomogram in the TCGA cohorts. (**E**) The calibration curves of nomogram in the ICGC cohorts.

To further improve the accuracy of prognostic predictions, univariate and multivariate Cox regression analyses were performed to analyze other potential variables in addition to risk scores, namely age, sex, grade, T stage, M stage, and clinical stage. The results indicated that the risk scores, age, grade, and M stage were independent prognostic factors for patients (Table 1). Subsequently, we developed a prognostic nomogram to predict OS in patients with KIRC. Risk scores, age, grade, and M stage were integrated into the nomogram (Figure 5A). The AUC values of this model for the prediction of 1-, 3-, and 5-year OS were 0.76, 0.77, and 0.78, respectively, in the TCGA cohort and 0.71, 0.70, and 0.75 in the ICGC cohort (Figure 5B, 5C). The calibration plots indicated that this model had good agreement between the prediction and real-life survival (Figure 5D, 5E).

**Table 1:** Univariable and multivariable analysis of the signature based on FOX genes and clinical factors in the TCGA cohort.

**Characteristic**	**Univariable analysis**		**Multivariable analysis**	
	**HR (95% CI)**	***p*-value**	**HR (95% CI)**	***p*-value**
Risk Score (High vs. Low)	2.8502 (2.0442–3.9741)	<0.0001	2.1014 (1.4907–2.9624)	<0.0001
Age (≥60 vs. <60)	1.7584 (1.2862–2.4039)	0.0004	1.5884 (1.1555–2.1834)	0.0002
Gender (Female vs. Male)	0.9331 (0.6797–1.2810)	0.6685	0.8486 (0.6124–1.1759)	0.3241
Grade (III/IV vs. I/II)	2.6387 (1.8637–3.7360)	<0.0001	1.5698 (1.0811–2.27964)	0.0178
Stage (III/IV vs. I/II)	3.7675 (2.7215-5.2155)	<0.0001	2.0852 (0.9077–4.3149)	0.0576
T (T3/4 vs. T1/2)	3.1139 (2.2808–4.2512)	<0.0001	0.8794 (0.4682–1.6514)	0.6893
M (M1 vs. M0)	4.3263 (3.1552–5.9321)	<0.0001	2.3598 (1.5986–3.4834)	<0.0001

### Correlation analyses of risk scores and immune response

**Figure 6:**
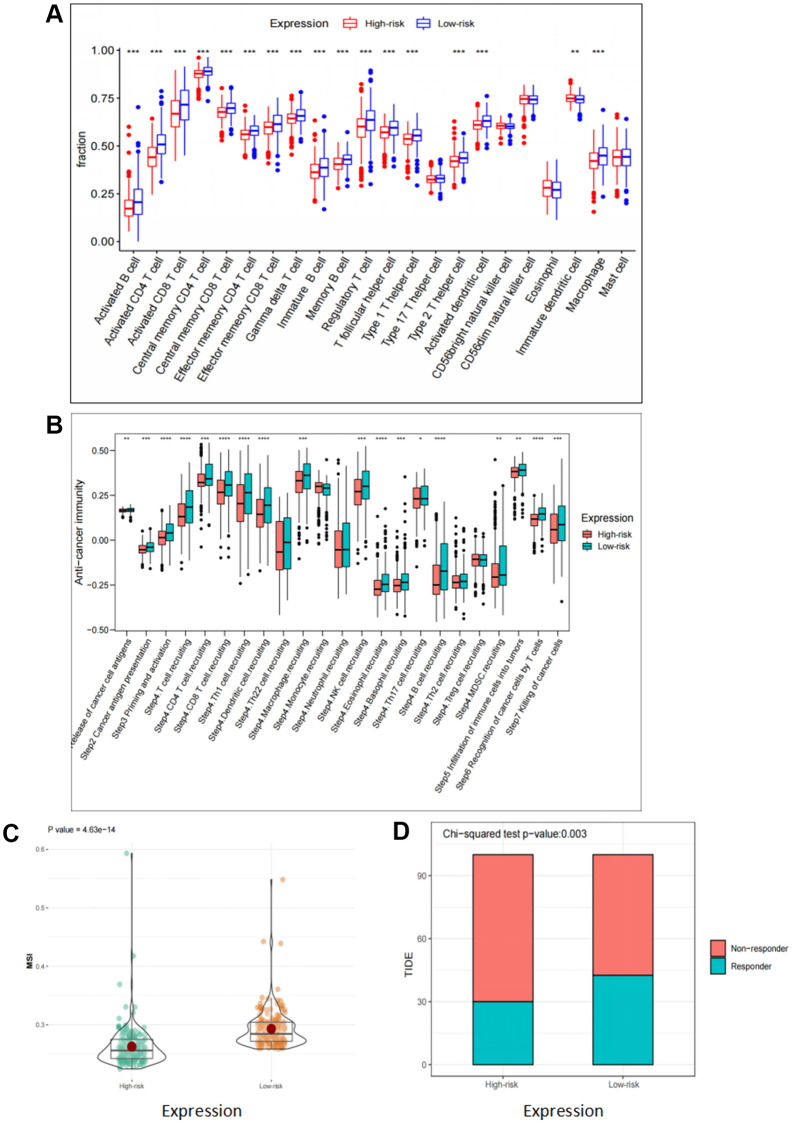
**Correlation analyses of risk scores and immune response.** (**A**) Comparison of 22 tumor-infiltrating immune cells in high risk and low risk group. (**B**) Differences in anti-cancer immunity cycle between high risk and low risk groups. (**C**, **D**) TIDE and MSI scores in high risk and low risk group.

Previous studies have revealed that besides their functions in regulating tumor development, FOX family genes also contribute to the regulation of the immune response [17]. Firstly, we assessed the correlation between these six FOX family genes and tumor immune cell infiltration. The results demonstrated that patients in the low-risk group had a higher infiltration levels of both innate and adaptive immune cells (Figure 6A). Next, we explored the association between the six FOX family genes and immune responses. The results demonstrated that every step in the cancer immunity cycle was downregulated in high-risk patients (Figure 6B). A higher TIDE and lower MSI score were found in the high-risk group, indicating a poor clinical immunotherapy efficacy (Figure 6C, 6D). These results show that the six prognosis-related FOX family genes we selected may play a vital role in predicting the efficacy of immunotherapy in KIRC patients.

### Expression and Kaplan-Meier survival analysis of the six FOX family genes

**Figure 7:**
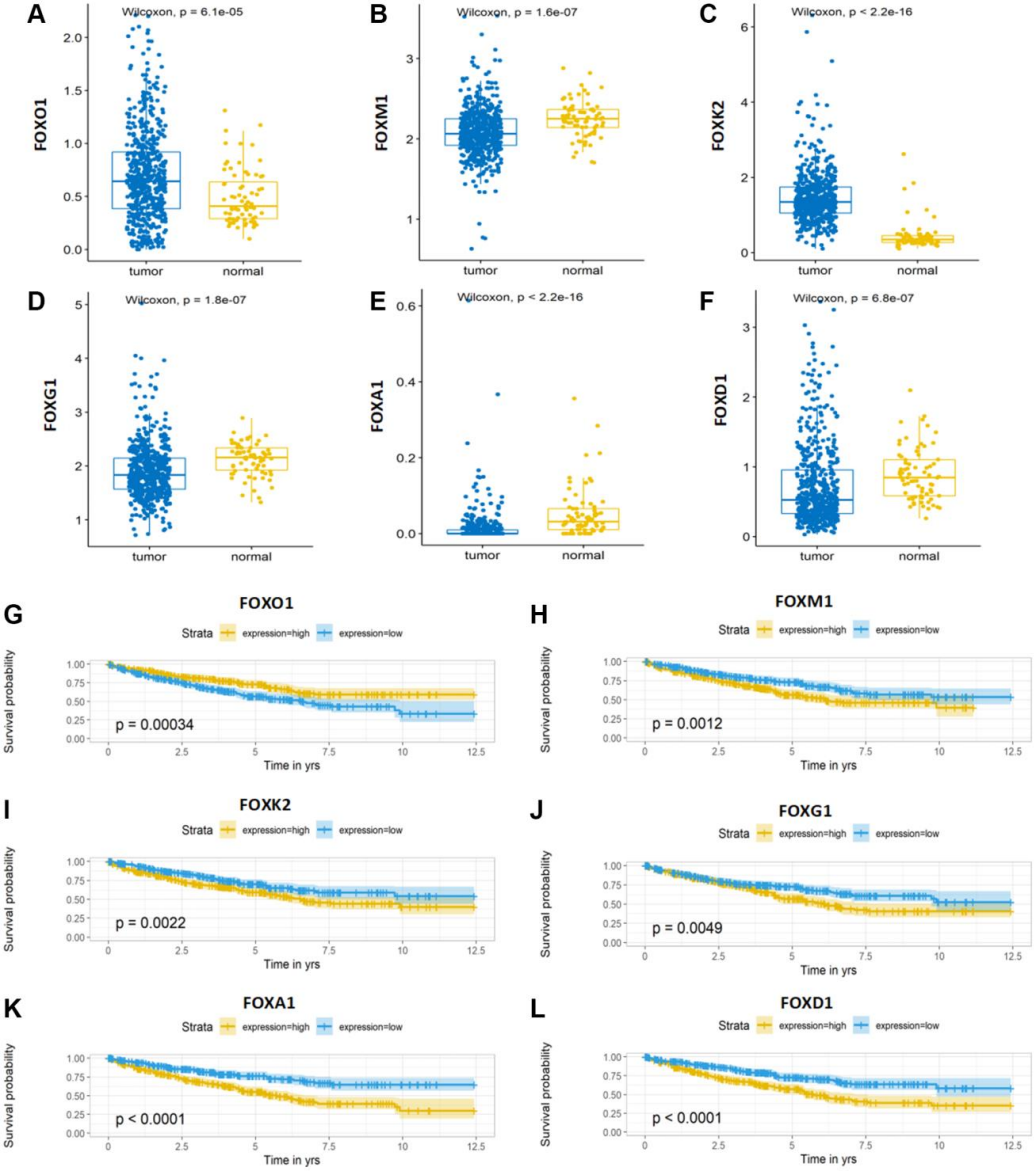
**Expression and Kaplan-Meier survival analysis of the six FOX family genes.** (**A**–**F**) A comparison of the expression levels of FOXO1, FOXM1, FOXK2, FOXG1, FOXA1 and FOXD1 in TCGA KIRC tumor tissue and adjacent normal tissue. (**G**–**L**) Kaplan-Meier curves for the six genes in the TCGA KIRC cohort.

To explore the effects of the six FOX family genes on the prognosis of patients with KIRC, we examined the expression of six FOX family genes in TCGA datasets. The expression of FOXO1 and FOXK2 in KIRC tumors was significantly lower than that in adjacent normal tissues, whereas the expression of FOXM1, FOXG1, FOXA1, and FOXD1 was significantly higher in tumor tissues (Figure 7A–7F). Moreover, Kaplan-Meier curves showed that higher expression of FOXM1, FOXK2, FOXG1, FOXA1, and FOXD1 was associated with poorer OS, whereas higher FOXO1 expression predicted a better OS (Figure 7G–7L).

### FOXG1 promotes proliferation of RCC cell lines

**Figure 8:**
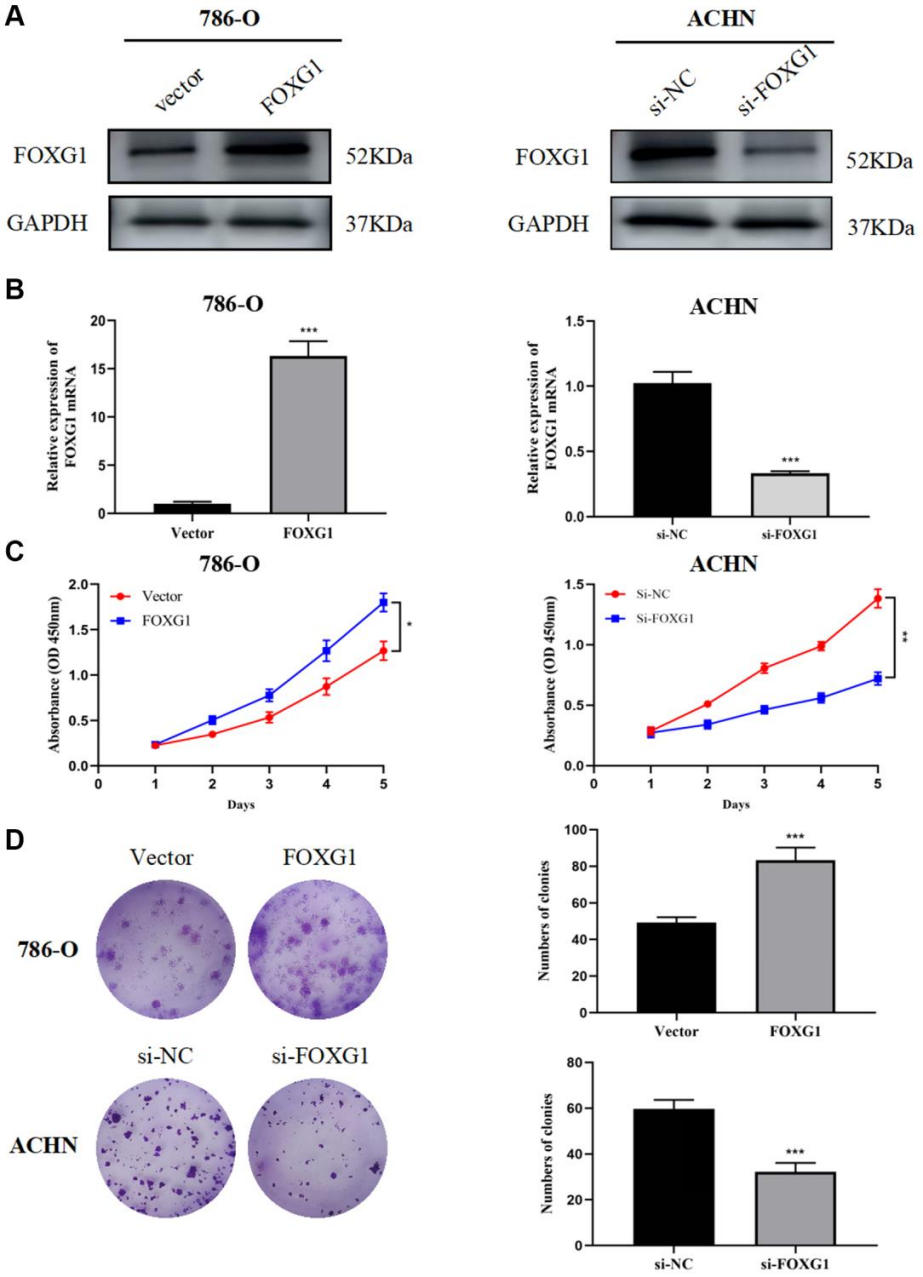
**FOXG1 promotes proliferation of RCC cell lines.** The efficiency of FOXG1 overexpression and knockdown was evaluated by western blot (**A**) and qRT-PCR (**B**) analyses. (**C**) Results of the CCK-8 assay on 786-O and ACHN cells following FOXG1 overexpression or silencing. (**D**) Results of colony formation assays on 786-O and ACHN cells after FOXG1 was overexpressed or knocked down. The data are presented as the mean ± standard deviation (SD) of experiments performed in triplicate. ^*^p < 0.05, ^**^p < 0.01, ^***^p < 0.001.

Through a literature search, we found that, except for FOXG1, the remaining five genes have been experimentally studied in RCC. In this research, a total of 82 surgically resected tumor tissues and matched normal kidney tissues from ccRCC patient were collected. The results showed that FOXG1 protein and mRNA levels were higher in tumor tissues than matched adjacent normal tissues. (Supplementary Figure 1A, 1B). Previous studies have indicated the oncogene role of FOXG1 in multiple invasive cancers such as glioblastoma, cutaneous squamous cell carcinoma, and cervical cancer [18–20]. However, the role of FOXG1 in KIRC remains to be elucidated. First, FOXG1 expression was upregulated in 786-O cells using a FOXG1-overexpression plasmid, whereas FOXG1 was silenced in ACHN cells using specific siRNAs. As shown in Figure 8A, 8B, western blotting and qRT-PCR analyses were performed to verify transfection efficiency.

To evaluate the role of FOXG1 in RCC proliferation, CCK-8 and colony formation assays were performed. The CCK-8 assay showed that FOXG1 silencing curbed ACHN cell proliferation, while FOXG1 overexpression promoted 786-O cell growth (Figure 8C). The results of colony formation assays further confirmed that downregulation of FOXG1 expression suppressed cell proliferation. FOXG1 therefore promoted tumor proliferation in RCC cells (Figure 8D).

### FOXG1 promotes migration and invasion of RCC cell lines

**Figure 9:**
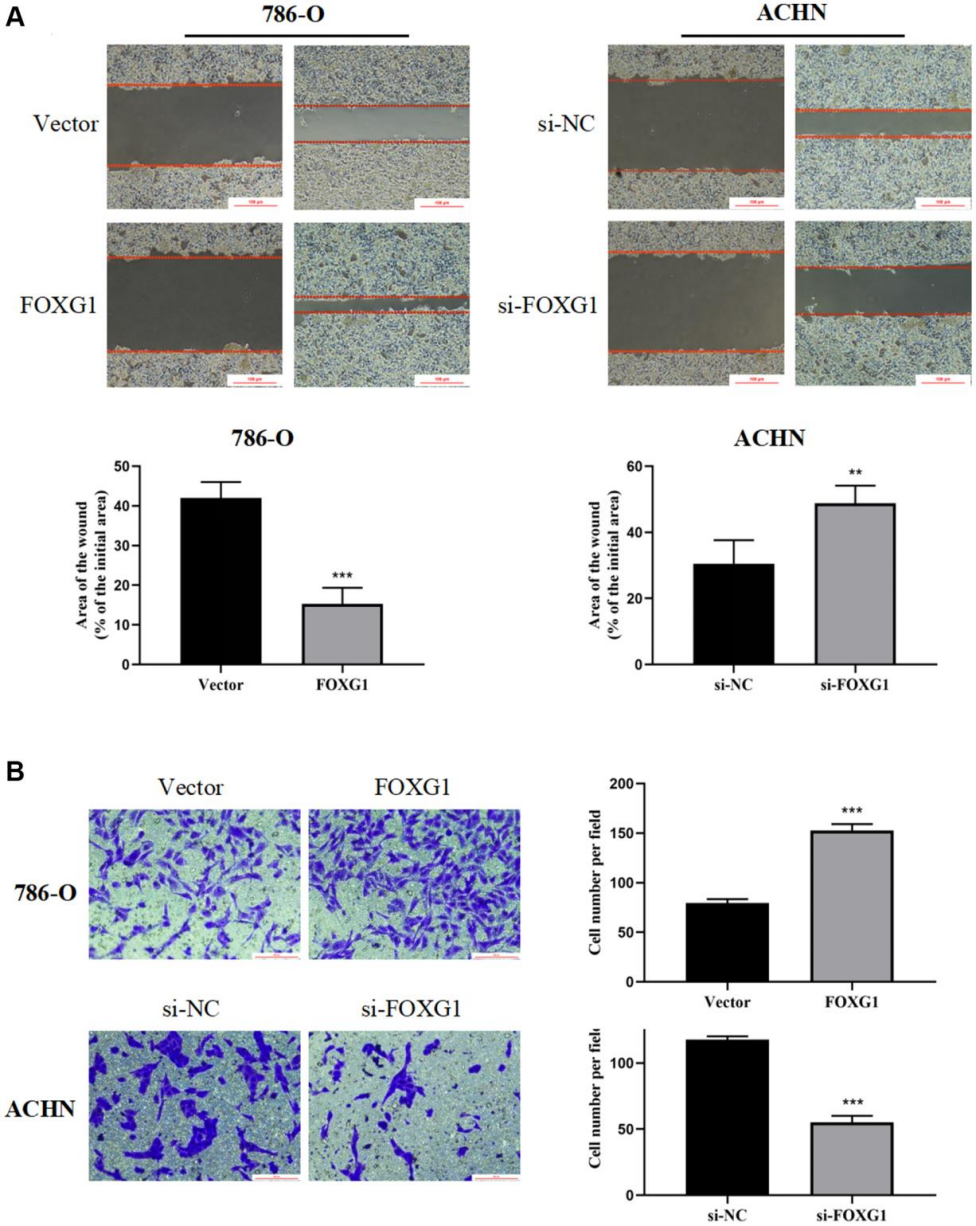
**FOXG1 promotes migration and invasion of RCC cell lines.** (**A**) Wound healing assay results of 786-O and ACHN cells after overexpression or knockdown of FOXG1. (**B**) Transwell assay result of 786-O and ACHN cells following FOXG1 overexpression or silencing. The data are presented as the mean ± standard deviation (SD) of experiments performed in triplicate. ^*^p < 0.05, ^**^p < 0.01, ^***^p < 0.001.

Next, we investigated whether FOXG1 influences the migration and invasion of RCC cells. The wound-healing assay showed that overexpression of FOXG1 enhanced the migration ability of 786-O cells. However, the downregulation of FOXG1 inhibited the migration of ACHN cells (Figure 9A). Concordant with these results, the transwell invasion assay also revealed that knockdown of FOXH1 impaired the invasive properties of ACHN cells, whereas FOXG1 overexpression promoted the invasive ability of 786-O cells (Figure 9B). Taken together, these data illustrate that FOXG1 enhances the migration and invasion of RCC cells.

## DISCUSSION

Renal cell cancer (RCC) is a malignant tumor with remarkable heterogeneity and complex pathogenesis. Currently, the development of bioinformatics and the publication of large sequencing cohort data have provided the basis for the study of RCC pathogenesis and prognosis model construction. Ning et al. [21]. detected hypoxia-related genes in KIRC using TCGA and ICGC datasets. A six hypoxia-related gene prognostic model was established to predict OS in patients with KIRC. Zheng et al. [22]. Also established a similar prognostic model for KIRC patients via comprehensive analysis of tripartite motif (TRIM) proteins. In this study, we screened potential FOX family genes that are closely related to the prognosis of KIRC and constructed a prognostic model. We then combined these genes with other clinical indicators to construct a nomogram model for KIRC to improve the predictive accuracy of the model. In addition, we performed an experimental study on FOXG1.

The FOX gene family plays a vital role in regulating proliferation, migration, and invasion of different types of cancers. It was also found that FOX family members are prone to drug resistance and stem cells [23]. Numerous FOX family members are abnormally expressed in RCC and may act as prognostic indicators of the disease. Univariate Cox regression analysis and screening of differentially expressed genes revealed that 17 genes were significantly differentially expressed and were strongly associated with KIRC prognosis. Based on this, the LASSO regression method and multivariate Cox regression analyses were used to identify a six-gene prognosis model to divide the patients into low- and high-risk groups. According to Kaplan-Meier curves, patients at high risk had worse OS. Time-dependent ROC analysis was used to verify the sensitivity and specificity of our prognostic signature based on six FOX gene prognosis models. The nomogram further improved mode accuracy by incorporating additional clinical information, including age, grade, clinical stage, and M stage. These results indicated that the nomogram we built was helpful in predicting patient survival and understanding how to manage patients with KIRC on a more personalized basis.

Immunotherapy, based on immune checkpoint inhibitors that target the cytotoxic T lymphocyte antigen-4 (CTLA-4) and programmed death-1 (PD-1)/programmed cell death-ligand 1 (PD-L1) axis, has been successfully applied and clinically verified in a fraction of KIRC patients. Currently, a major obstacle to the widespread application of immunotherapy is the precise selection of patients who will benefit from this treatment. Predictive biomarkers, including PD-1/PD-L1 expression level, microsatellite instability, tumor mutation burden, and monocyte-to-lymphocyte ratio, have been proposed to be correlated with progression-free survival and overall survival [24]. Additionally, the quantity and composition of tumor-infiltrating immune cells (TIICs) are considered to be the new underpinnings of immunotherapy [25, 26]. In our study, we found that there were significant differences in immune status and immune cell infiltration proportions between the high- and low-risk groups. There was high infiltration of adaptive and innate immune cells in the low-risk group. Furthermore, compared to the low-risk group, the anticancer immune response might be significantly suppressed in the high-risk group.

The prognosis model was comprised of six FOX family genes: FOXO1, FOXM1, FOXK2, FOXG1, FOXA1, and FOXD1. Among them, only high FOXO1 expression was associated with the low-risk group, and the expression of the remaining five genes was positively associated with the high-risk group. There was a significant decrease in FOXO1 expression in KIRC tissues compared to that in non-tumor renal tissues at the RNA and protein levels. Downstream of multiple oncogenes and tumor suppressor genes, decreased FOXO1 expression promotes tumor metastasis and is positively correlated with poor survival outcomes [27, 28]. FOXK2 inhibits the proliferation, migration, and invasive ability of ccRCC cells and induces apoptosis *in vitro* by interacting with the potential downstream gene epidermal growth factor receptor (EGFR) [29]. In addition, FOXK2 acts as an important regulator of cellular metabolism to induce aerobic glycolysis and inhibit the reduction of pyruvate to lactate [30]. However, in the present model, FOXK2 remained positively associated with patients in the high-risk group; further studies are needed to support the relationship between FOXK2 and prognosis. Previous studies have also demonstrated that FOXA1 and FOXD1 are broadly involved in tumor development as well as mitochondrial metabolism. Silencing FOXA1 or FOXD1 in RCC inhibits tumor growth by inhibiting cell cycle progression [31, 32]. Moreover, recent research has indicated that FOXA1 may participate in sunitinib resistance in RCC [33]. Several studies have confirmed the vital role of FOXM1, which may serve as an essential prognostic biomarker and therapeutic target for renal cancer. MicroRNA-320a and pre-miR-149 serve as antitumor miRNAs by downregulating FOXM1 [34, 35]. As transcription factors, FOXM1 can regulate downstream cell cycle-related genes, including PLK, cyclin B1, cyclin D1, and Cdk2, to promote cell proliferation and tumor progression [36]. Considering the lack of experimental studies on the role of FOXG1 in kidney cancer, we conducted several experiments and confirmed that FOXG1 promotes cell growth and invasion *in vitro*.

This study has a few limitations. First, the analytical data were derived from TCGA and ICGC databases, and to clarify the accuracy of the model in predicting survival, we need to validate it based on real-world clinical data. Further *in vivo* and *in vitro* verification experiments are required to validate the role and mechanism of FOX family genes in KIRC. What’s more, artificial intelligence is currently used to improve the prediction efficacy of conventional statistical analysis and nomograms and is applied in the field of urology as well as for the prediction of recurrence or overall survival after surgery. Chan X, et al. constructed and validated a clinical prediction model for lung metastasis in renal cancer patients based on AI algorithm. The AUC of ranked from 0.907 to 0.934, which revealed the high applicability of the model [37]. We will include more patient data from the real world and compare machine learning algorithms with traditional algorithms in later studies.

In conclusion, we comprehensively examined the relationship between FOX expression and survival in patients. An independent prognosis model based on the six FOX genes has been developed to predict the prognosis of KIRC patients. Furthermore, experimental evidence indicates that FOXG1 promotes KIRC progression, which fills a research gap and strengthens the rationality of our prognostic model.

## Supplementary Materials

Supplementary Figure 1

Supplementary Table 1
