# Supplementary material for: A comprehensive analysis of the FOX family for predicting kidney renal clear cell carcinoma prognosis and the oncogenic role of FOXG1
Source: Aging (Albany NY). 2022 Dec 29;14(24):10107–24. doi: 10.18632/aging.204448 (PMC9831721; doi:10.18632/aging.204448)
Supplement: Supplementary Figure 1 [file aging-14-204448-s001.pdf]

SUPPLEMENTARY FIGURE

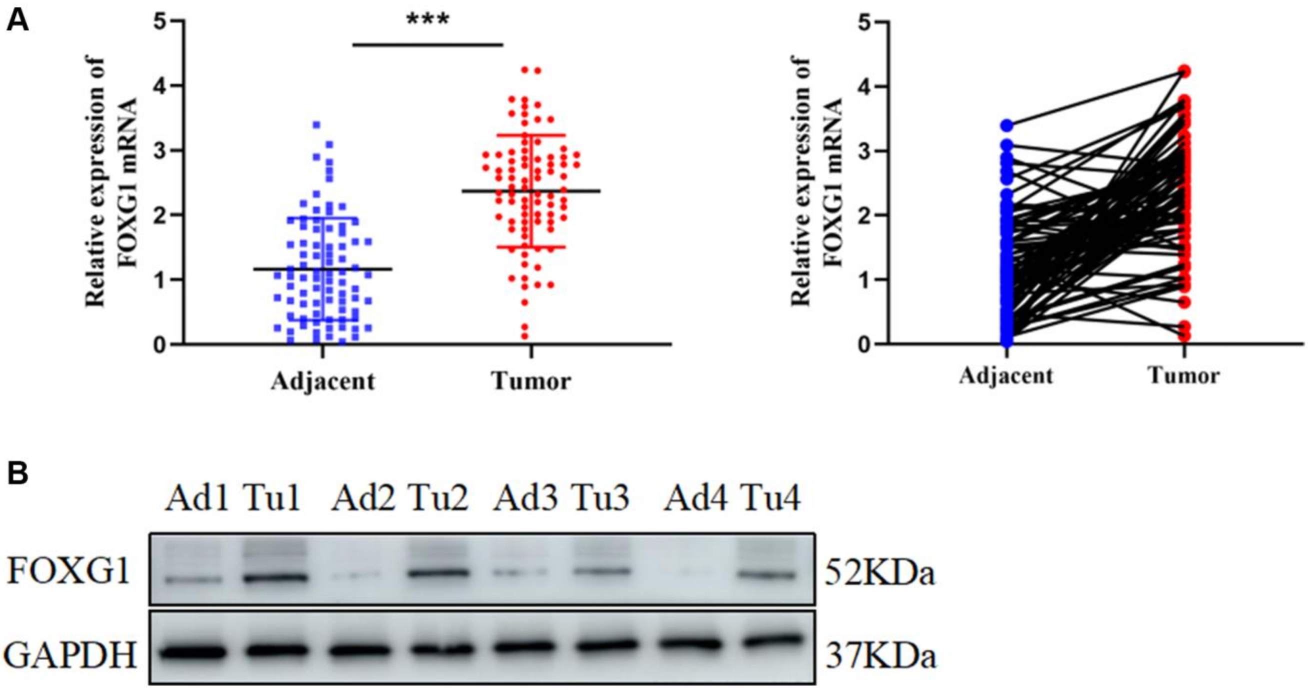

Supplementary Figure 1. FOXG1 expression is significantly up-regulated in ccRCC tissues. FOXG1 mRNA (**A**) and protein (**B**) levels in ccRCC tumor tissues and corresponding adjacent nontumor tissues. $^{*}p < 0.05$, $^{**}p < 0.01$, $^{***}p < 0.001$.
