# Supplementary material for: A comprehensive analysis of the FOX family for predicting kidney renal clear cell carcinoma prognosis and the oncogenic role of FOXG1
Source: Aging (Albany NY). 2022 Dec 29;14(24):10107–24. doi: 10.18632/aging.204448 (PMC9831721; doi:10.18632/aging.204448)
Supplement: Supplementary Table 1 [file aging-14-204448-s002.pdf]

SUPPLEMENTARY TABLE

Supplementary Table 1. Univariable analysis of different expressed FOX genes in KIRC.

Gene names	Univariable analysis	
	HR (95% CI)	*p*-value
FOXA1	1.7176 (1.3799–2.1380)	<0.0001
FOXB2	0.0127 (0.0002–0.8065)	0.0392
FOXD1	1.5102 (1.2266–1.8594)	<0.0001
FOXD4L1	7.1292 (3.3840–26.7584)	<0.0001
FOXE1	2.3468 (1.6004–3.4412)	<0.0001
FOXE3	11.4500 (7.8558–43.6767)	<0.0001
FOXF2	1.5777 (1.0656–2.3358)	0.0227
FOXG1	1.6476 (1.0749–2.5253)	<0.0001
FOXH1	6.3034 (2.3373–16.9996)	0.0002
FOXK2	0.5133 (0.3877–0.7784)	<0.0001
FOXL1	1.4302 (1.1063–1.8490)	0.0063
FOXM1	1.9101 (1.6329–2.2341)	<0.0001
FOXN2	0.6511 (0.4902–0.8647)	0.0033
FOXO1	0.5653 (0.4365–0.7322)	<0.0001
FOXO4	0.5213 (0.3850–0.7058)	<0.0001
FOXP3	1.5007 (1.2248–1.8388)	<0.0001
FOXP4	1.8059 (1.3441–2.4263)	<0.0001
